# Arabidopsis ICS1 produces salicylic acid precursor isochorismate through a gated channel mechanism

**DOI:** 10.1093/plphys/kiae342

**Published:** 2024-06-14

**Authors:** Jiawen Chen

**Affiliations:** Assistant Features Editor, Plant Physiology, American Society of Plant Biologists; Division of Crop Biotechnics, Department of Biosystems, KU Leuven 3001 Leuven, Belgium

**Figure 1.**
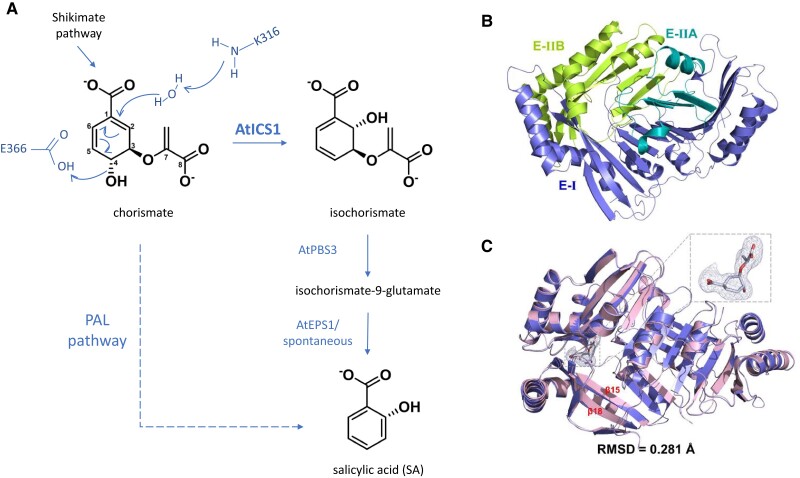
**A)** Schematic summarizing the 2 SA biosynthesis pathways in Arabidopsis, where AtICS1 facilitates the main pathway. The schematic also includes the general acid-general base mechanism of the AtICS1 reaction, drawn after the general ICS mechanism illustrated in Shelton and Lamb (2018). **B)** Crystal structure of AtICS1, colored by structural domains. The 2 main structural domains are E-I and E-II (with 2 subdomains), forming the 2 sheets of the β-sandwich, with the active site pocket at their interface, as shown in C. **C)** Superimposition of apo (slate blue) and chorismate-bound (pink) AtICS1 structures, with chorismate visualized within the catalytic site, with its polder omit map contoured at 3 σ (light blue mesh). RMSD (root mean square deviation) for the apo and holo structures is indicated. Figures B and C taken from Su et al. 2024.

When one part of a plant is under attack from pathogens, an extensive network of defense responses is triggered, signaling to other parts of the plant to prepare itself against the infection. This process is called systemic acquired resistance (SAR), and the hormone salicylic acid (SA) is essential for SAR signaling as well as local defense responses (Peng et al. 2021). In plants, 2 SA biosynthesis pathways have been identified: the isochorismate synthase (ICS) and phenylalanine ammonia lyase (PAL) pathways (Fig. 1). Both pathways use chorismate as a precursor, which is produced from the shikimate pathway. The 2 SA biosynthesis pathways have varying contributions depending on the species, and in Arabidopsis 90% of the defense-related SA production is through the ICS pathway (Garcion et al. 2008; Rekhter et al. 2019).

Arabidopsis has 2 ICS genes: *ICS1* and *ICS2* (Wildermuth et al. 2001). The ICS proteins localize to the chloroplast, and SA biosynthesis during defense responses is mainly carried out by AtICS1 (Garcion et al. 2008), proposed to be favored through transcriptional regulation (Wildermuth et al. 2001). AtICS1 is an isomerase that converts chorismate to isochorismate by changing a C4 hydroxyl group to C2. Isochorismate is then transported to the cytosol, where PBS3 converts it to isochorismate-9-glutamate, which is converted to SA through EPS1 or through spontaneous decay (Rekhter et al. 2019; Fig. 1). In addition to SA biosynthesis, isochorismate can also be a precursor for phylloquinone (Yokoo et al. 2018), an electron acceptor in the Photosystem I complex, placing isochorismate synthesis into a larger metabolic context.

ICS enzymes are part of the MST (manquinone, siderophore, and tryptophan) family of enzymes. These are magnesium-dependent enzymes that convert chorismate into various compounds and are well studied in bacteria (Shelton and Lamb 2018). The overall structure consists of a β-sandwich made up of 2 β-sheets and surrounding helices (Fig. 1), with an active site that sits between the 2 sheets and where a Mg^2+^ ion aids in a general acid-general base mechanism (Fig. 1). In bacteria, isochorismate is also converted to SA, but it is used to produce siderophores, which are iron chelators. *Escherichia coli* has 2 ICSs: EntC is involved in the production of siderophores and MenF produces menaquinones, electron acceptors. The structures for EntC and MenF are known (Parsons et al. 2008; Sridharan et al. 2010). Although previous biochemical studies and homology modelling showed there were likely similarities between bacterial and plant ICSs (Macaulay et al. 2017; Yokoo et al. 2018), no structures of plant ICSs had yet been determined. It was not known exactly how AtICS structure and catalytic activity are connected to its role in SA biosynthesis.

In this issue of *Plant Physiology*, Su et al. (2024) present the crystal structures of apo and chorismate-bound AtICS1, providing a comprehensive view of ICS activity in plants. They show that the overall mechanism is similar to what we predict from bacteria but that there are species-specific residues in the catalytic site that make a difference for catalytic efficiency. They purified recombinant AtICS1 and AtICS2 from *E. coli*, as well as ICS enzymes from soybean (*Glycine max*), tomato (*Solanum lycopersicum*), *Nicotiana benthamiana*, and rice (*Oryza sativa*). Comparative in vitro activity assays revealed different catalytic efficiencies and optimal concentrations of magnesium for the different ICS enzymes. Strikingly, AtICS1 and AtICS2 had similar catalytic activity, consistent with previous findings that their difference lies mainly in their expression patterns.

The newly obtained crystal structures of AtICS1 showed that it has a conserved topology consistent with other MST enzymes (Fig. 1), with a cavity around the catalytic site consisting of a channel and catalytic pocket, which can adopt both an open and closed conformation. Substrate binding does not lead to substantial conformational changes, but there are localized changes around the chorismate binding site (Fig. 1). Hydrogen bonding and hydrophobic interactions are important for substrate binding, with K316 identified as the general base that activates water molecules for nucleophilic attack at C2 and E366 as the general acid, which facilitates hydroxyl cleavage at C4 (Fig. 1). Mutating any of the amino acids interacting with chorismate resulted in a near or complete knockout of AtICS1 activity.

The results suggest that AtICS1 isomerization activity is regulated by a gating mechanism in an area between the substrate entrance channel and active site and by Mg^2+^ binding in the active site. Docking studies suggest that chorismate binds transiently in the substrate entrance channel through hydrophobic interactions, which could enhance the efficiency of substrate delivery. In the apo structure, AtICS1 has a higher binding affinity for chorismate in the channel; in the chorismate-bound holo form, AtICS1 has a higher affinity for chorismate binding in the catalytic site, suggesting the channel helps to bring chorismate to the catalytic site. By comparing AtICS1 with the known structures of EcMentF and EcEntC, the authors showed that binding of Mg^2+^ in the catalytic site coordinates the action of 2 essential glutamate residues, which facilitates the closing of the gate and optimizes AtICS1 catalytic activity. The authors propose an elegant model that consists of substrate delivery, closing of the channel-catalytic site gate (facilitated by Mg^2+^), isomerization of chorismate, and opening of the gate and release of the isochorismate product.

The controlled gating mechanism seems conserved between ICS enzymes from bacteria and other plants, but the specific efficiencies of the enzymes vary, depending on the active residues. For instance, comparing AtICS1 channel residues with OsICS1 residues demonstrated that a specific set of AtICS1 residues increases the catalytic efficiency of OsICS1 when all are mutated together. The authors also showed general variation in both the catalytic efficiencies of different ICS enzymes and their optimal Mg^2+^ concentrations, with higher optimal Mg^2+^ concentrations correlating with a higher catalytic activity.

Su et al. (2024) have provided a detailed mechanism of AtICS1 isomerization activity, using comparative enzymatic assays, structural analysis with X-ray crystallography, and mutagenesis studies. By including purified ICS enzymes from 4 different plants in addition to Arabidopsis, they have shed light on both the similarities and subtle but important differences between orthologs. This study improves our fundamental understanding of plant ICS biochemical mechanisms and invites further investigation into the diversity of ICS biochemistry and regulation in different species.
